# Supplementary material for: Establishment of an Antimicrobial Stewardship Program to Spare the Use of Oral Fluoroquinolones for Acute Uncomplicated Cystitis in Outpatients
Source: Antibiotics (Basel). 2024 Sep 14;13(9):886. doi: 10.3390/antibiotics13090886 (PMC11429053; doi:10.3390/antibiotics13090886)
Supplement: Supplementary file 1 [file antibiotics-13-00886-s001.zip › antibiotics-3182639-supplementary.pdf]

Supplementary Materials

Table S1. Total number of patients treated with fluoroquinolones and the number of days of administration (2020-2022).

**Days of administration per prescription, days**	**1–7**	**8–14**	**15–21**	**22–28**	**≥29**
Total number of patients treated with fluoroquinolones, n	5087	790	168	173	266
CPFX	329	19	25	1	21
LVFX	4573	596	113	88	145
MFLX	49	100	0	3	0
GRNX	77	17	2	1	7
TFLX	23	7	1	1	0
STFX	36	51	27	79	93
Days of administration					
Average, days	5	12	20	27	56
Median (IQR), days	5 (3–7)	11 (10-14)	21 (18–21)	28 (28)	49 (41-84)
Estimated ratio†	1	2	4	6	12
Estimated number of prescriptions‡ (estimated percentage)	5,087 (44.0%)	1,580 (13.7%)	672 (5.8%)	1,038 (9.0%)	3,192 (27.5%)

† Calculated based on group with prescription period of 1-7 days average numbers. ‡ Calculated by multiplying the total number of patients treated with fluoroquinolones by the estimated ratio. Abbreviations: CPFX, ciprofloxacin; LVFX, levofloxacin; MFLX, moxifloxacin; GRNX, garenoxacin mesilate hydrate; TFLX, tosufloxacin; STFX, sitafloxacin; IQR, interquartile range.

Table S2. Number of fluoroquinolone prescriptions in each clinical department.

**Clinical department**	**Number of fluoroquinolone prescriptions, n**
Urology	179
General medical department	67
Obstetrics and gynecology	15
Nephrology	12
Pediatrics	10
Oncology	4
Gastroenterology	4
Neurology	3
Orthopedic surgery	3
Rheumatology	2
Cardiology	2
Ophthalmology	1
Cardiovascular surgery	1
Respiratory medicine	1
Breast surgery	1
